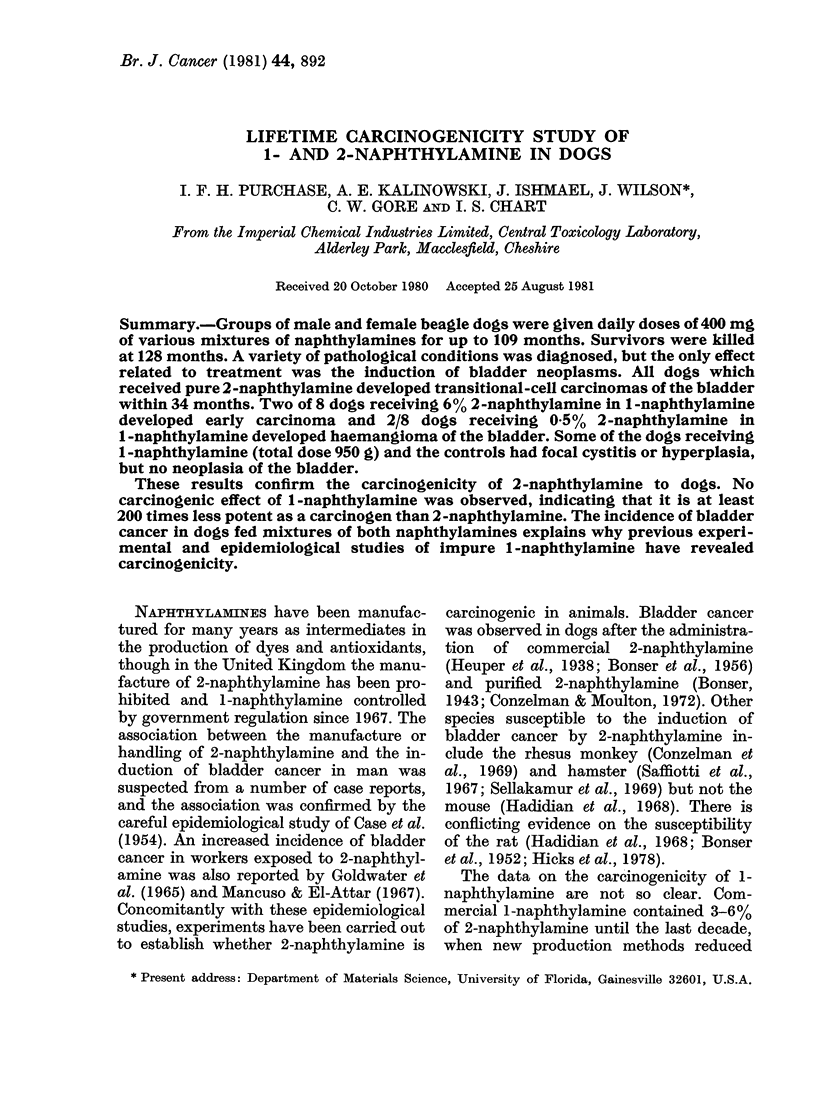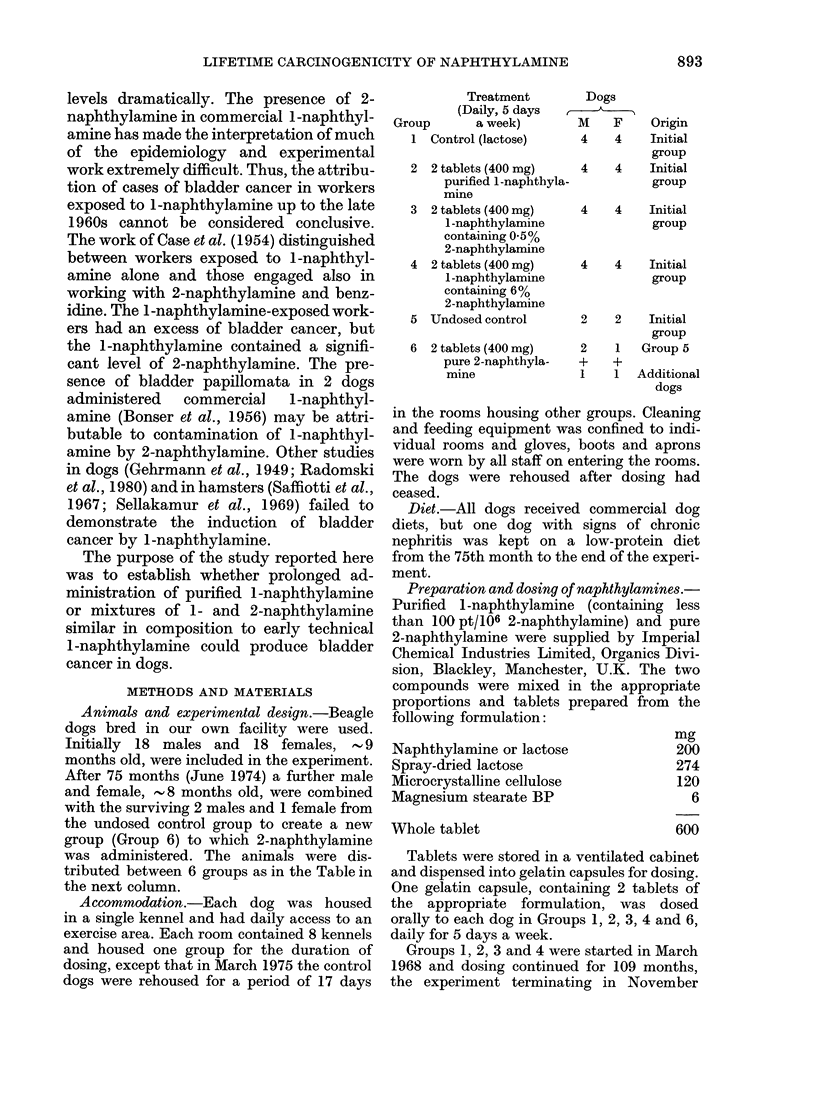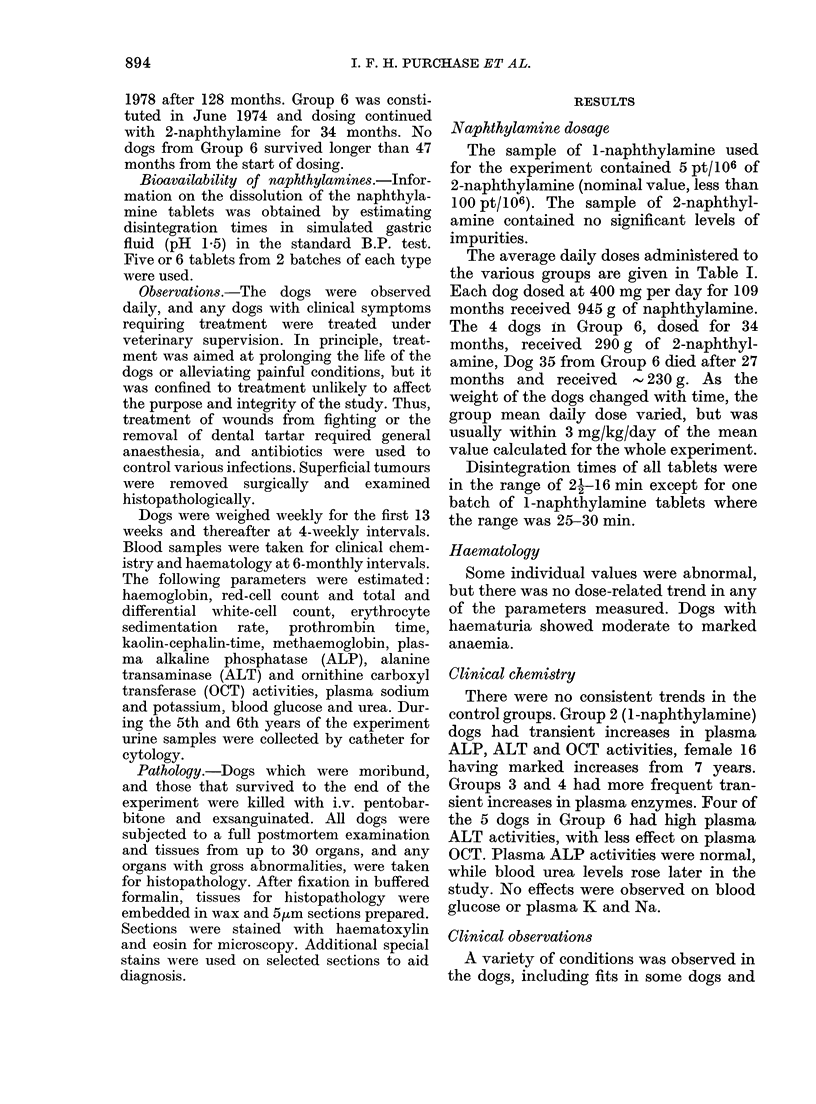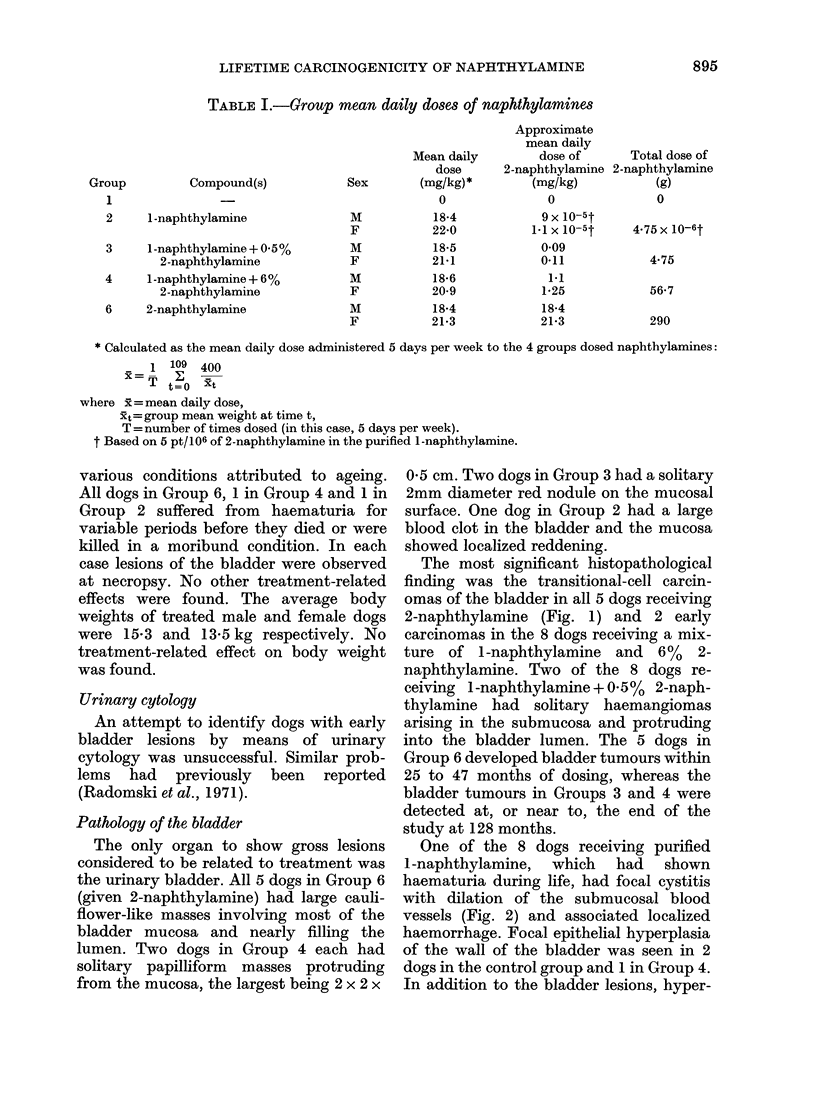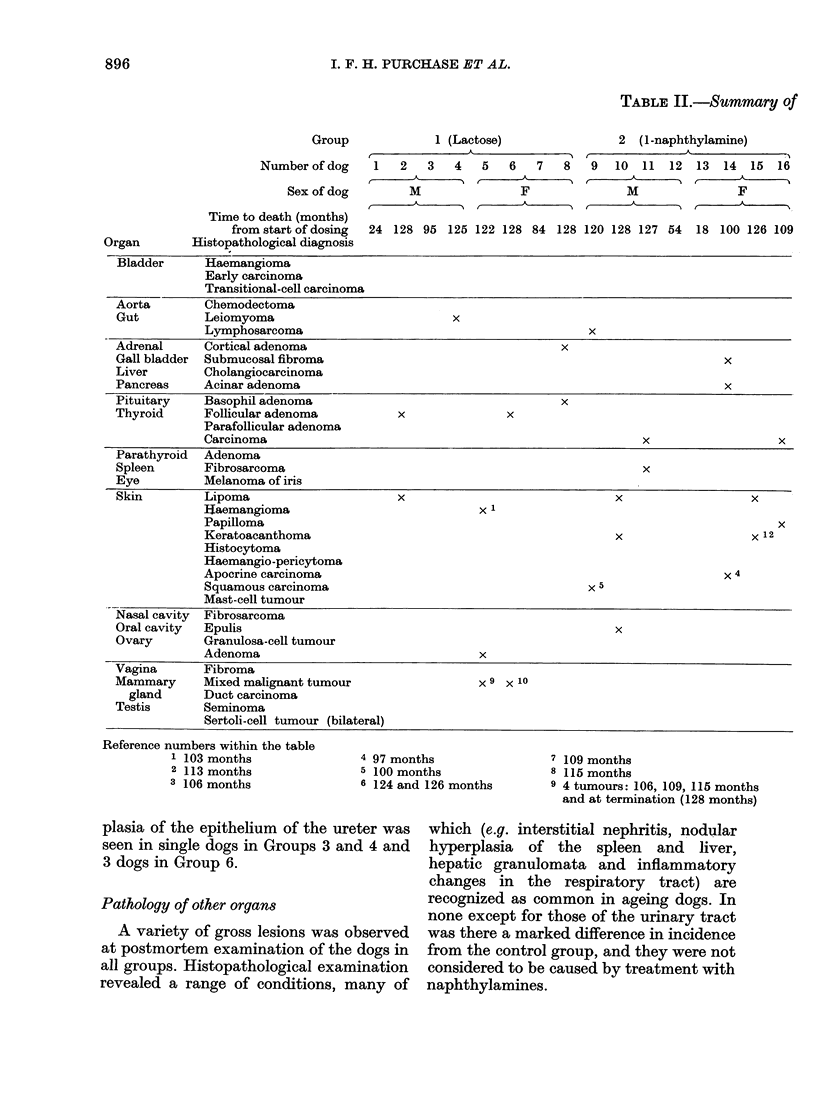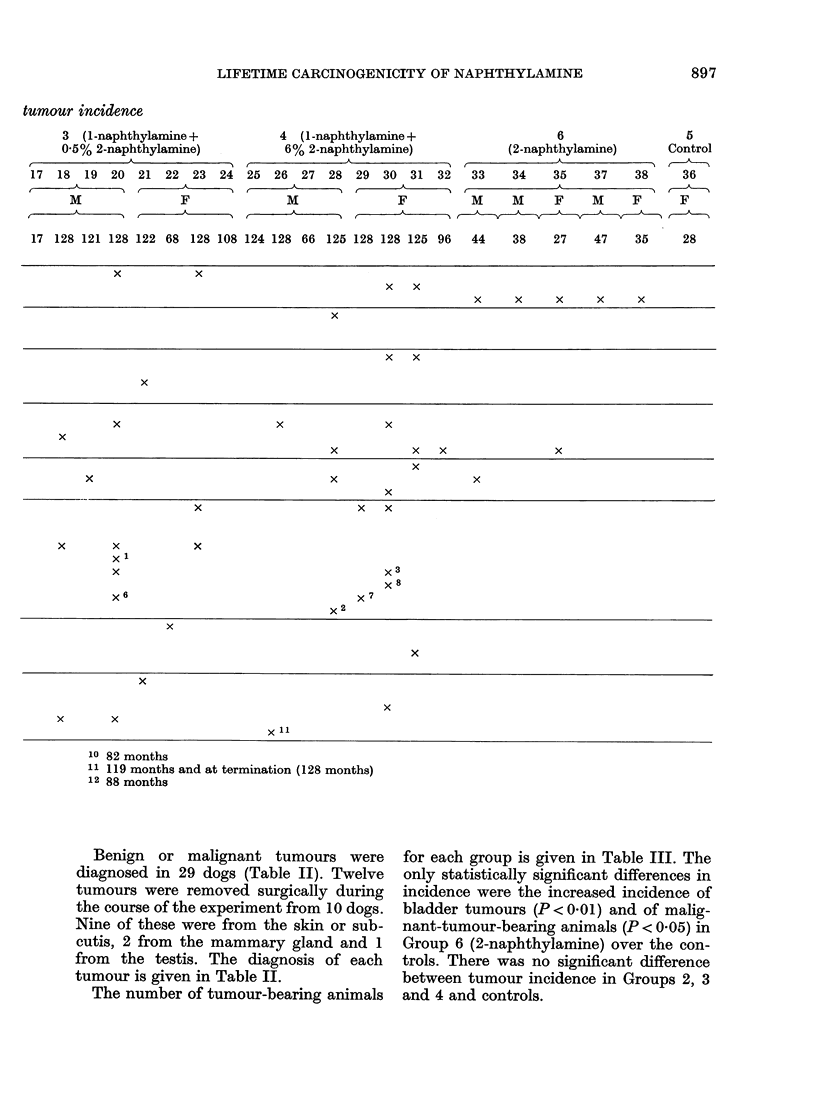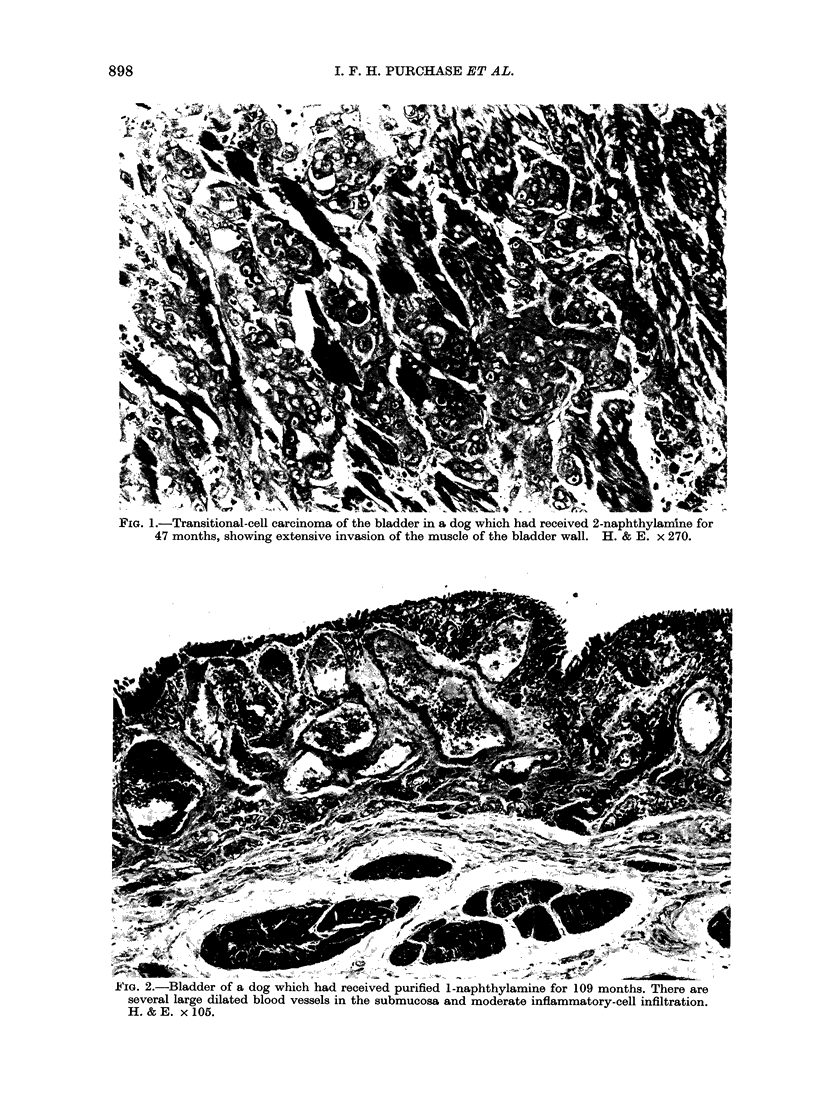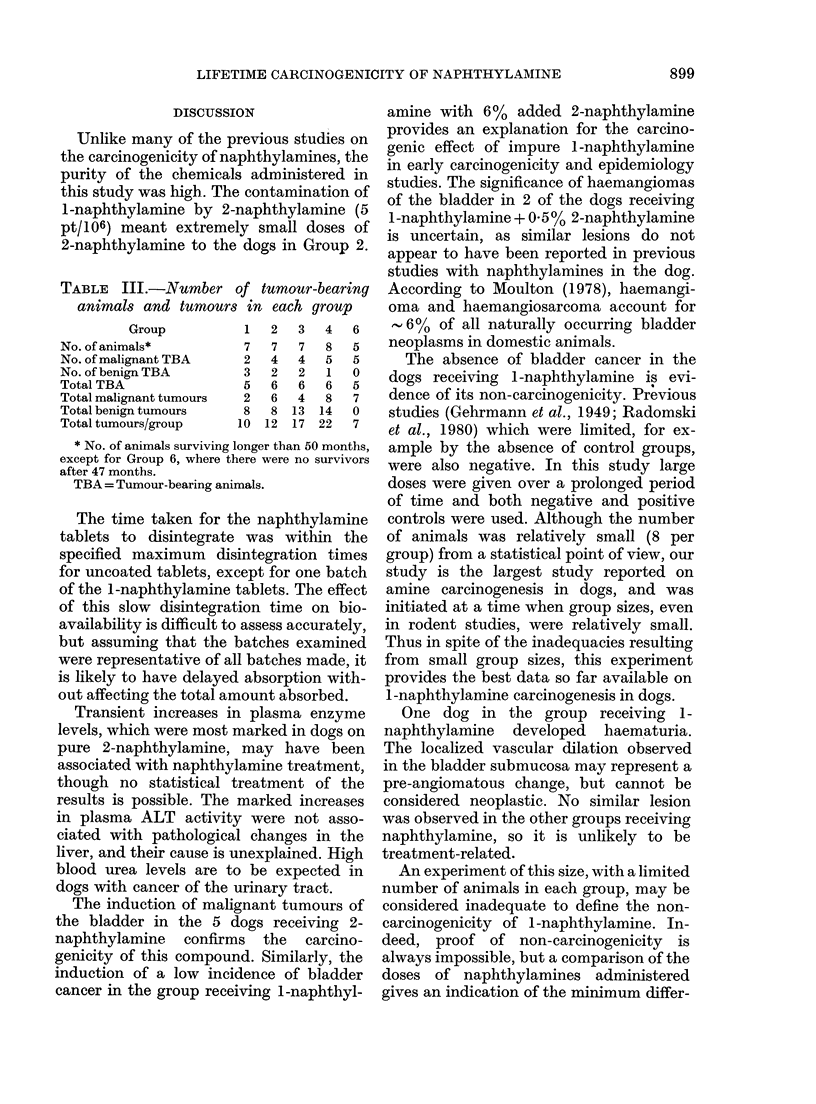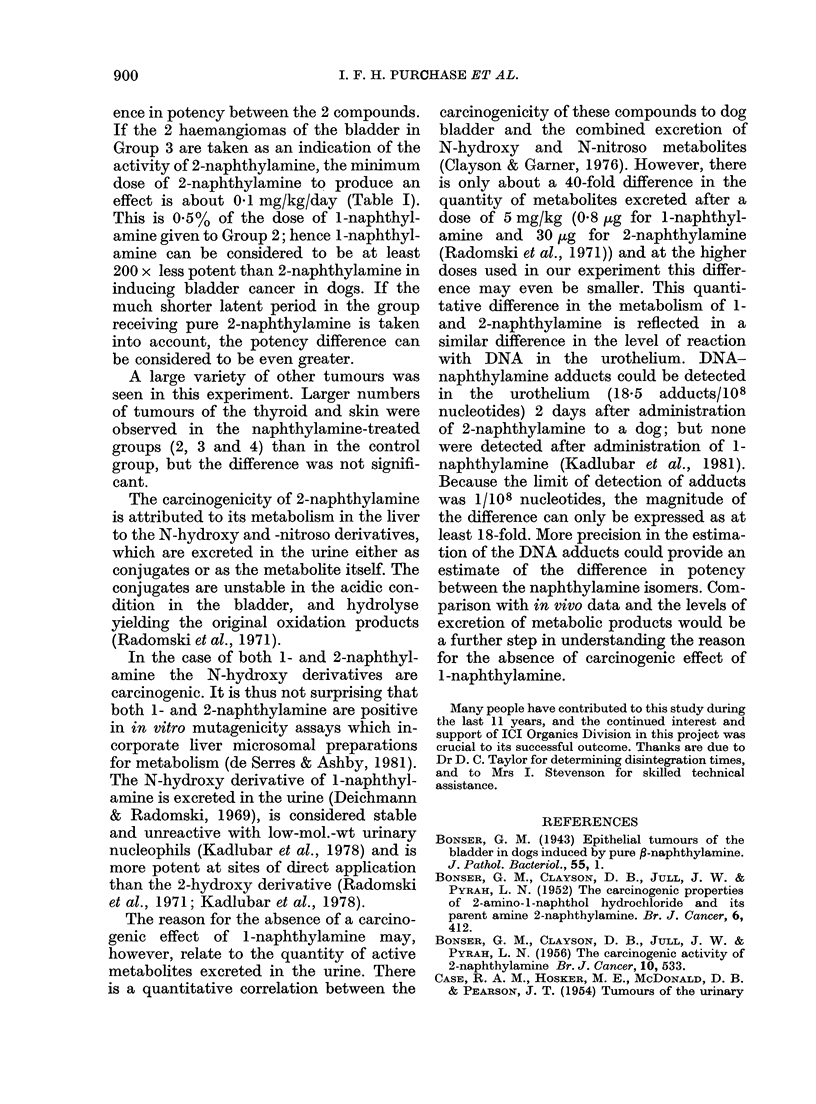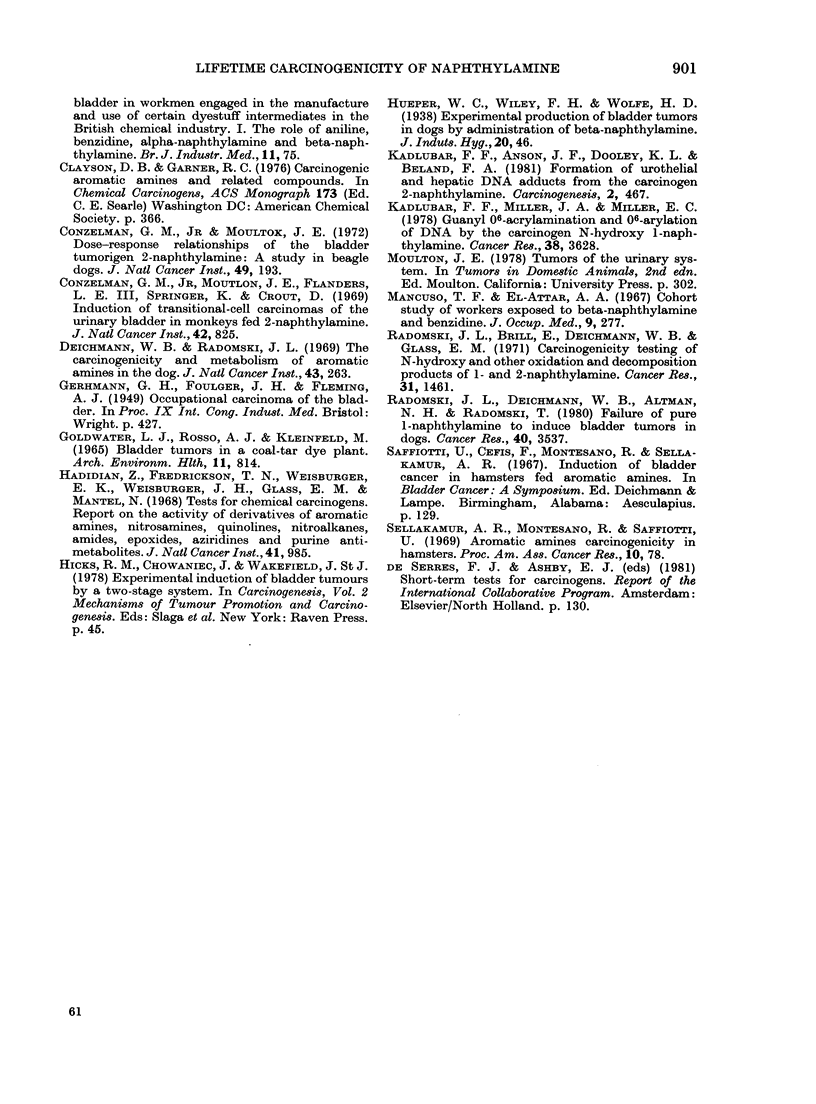# Lifetime carcinogenicity study of 1- and 2-naphthylamine in dogs.

**DOI:** 10.1038/bjc.1981.289

**Published:** 1981-12

**Authors:** I. F. Purchase, A. E. Kalinowski, J. Ishmael, J. Wilson, C. W. Gore, I. S. Chart

## Abstract

**Images:**


*Br. J. Cancer* (1981) **44**, 892

### LIFETIME CARCINOGENICITY STUDY OF 1- AND 2-NAPHTHYLAMINE IN DOGS

I. F. H. PURCHASE, A. E. KALINOWSKI, J. ISHMAEL, J. WILSON*, C. W. GORE AND I. S. CHART

*From the Imperial Chemical Industries Limited, Central Toxicology Laboratory, Alderley Park, Macclesfield, Cheshire*

Received 20 October 1980 Accepted 25 August 1981

**Summary.—Groups of male and female beagle dogs were given daily doses of 400 mg of various mixtures of naphthylamines for up to 109 months. Survivors were killed at 128 months. A variety of pathological conditions was diagnosed, but the only effect related to treatment was the induction of bladder neoplasms. All dogs which received pure 2-naphthylamine developed transitional-cell carcinomas of the bladder within 34 months. Two of 8 dogs receiving 6% 2-naphthylamine in 1-naphthylamine developed early carcinoma and 2/8 dogs receiving 0·5% 2-naphthylamine in 1-naphthylamine developed haemangioma of the bladder. Some of the dogs receiving 1-naphthylamine (total dose 950 g) and the controls had focal cystitis or hyperplasia, but no neoplasia of the bladder.**

**These results confirm the carcinogenicity of 2-naphthylamine to dogs. No carcinogenic effect of 1-naphthylamine was observed, indicating that it is at least 200 times less potent as a carcinogen than 2-naphthylamine. The incidence of bladder cancer in dogs fed mixtures of both naphthylamines explains why previous experimental and epidemiological studies of impure 1-naphthylamine have revealed carcinogenicity.**

NAPHTHYLAMINES have been manufactured for many years as intermediates in the production of dyes and antioxidants, though in the United Kingdom the manufacture of 2-naphthylamine has been prohibited and 1-naphthylamine controlled by government regulation since 1967. The association between the manufacture or handling of 2-naphthylamine and the induction of bladder cancer in man was suspected from a number of case reports, and the association was confirmed by the careful epidemiological study of Case *et al.* (1954). An increased incidence of bladder cancer in workers exposed to 2-naphthylamine was also reported by Goldwater *et al.* (1965) and Mancuso & El-Attar (1967). Concomitantly with these epidemiological studies, experiments have been carried out to establish whether 2-naphthylamine is carcinogenic in animals. Bladder cancer was observed in dogs after the administration of commercial 2-naphthylamine (Heuper *et al.*, 1938; Bonser *et al.*, 1956) and purified 2-naphthylamine (Bonser, 1943; Conzelman & Moulton, 1972). Other species susceptible to the induction of bladder cancer by 2-naphthylamine include the rhesus monkey (Conzelman *et al.*, 1969) and hamster (Saffiotti *et al.*, 1967; Sellakamur *et al.*, 1969) but not the mouse (Hadidian *et al.*, 1968). There is conflicting evidence on the susceptibility of the rat (Hadidian *et al.*, 1968; Bonser *et al.*, 1952; Hicks *et al.*, 1978).

The data on the carcinogenicity of 1-naphthylamine are not so clear. Commercial 1-naphthylamine contained 3–6% of 2-naphthylamine until the last decade, when new production methods reduced

* Present address: Department of Materials Science, University of Florida, Gainesville 32601, U.S.A.

levels dramatically. The presence of 2-naphthylamine in commercial 1-naphthylamine has made the interpretation of much of the epidemiology and experimental work extremely difficult. Thus, the attribution of cases of bladder cancer in workers exposed to 1-naphthylamine up to the late 1960s cannot be considered conclusive. The work of Case *et al.* (1954) distinguished between workers exposed to 1-naphthylamine alone and those engaged also in working with 2-naphthylamine and benzidine. The 1-naphthylamine-exposed workers had an excess of bladder cancer, but the 1-naphthylamine contained a significant level of 2-naphthylamine. The presence of bladder papillomata in 2 dogs administered commercial 1-naphthylamine (Bonser *et al.*, 1956) may be attributable to contamination of 1-naphthylamine by 2-naphthylamine. Other studies in dogs (Gehrmann *et al.*, 1949; Radomski *et al.*, 1980) and in hamsters (Saffiotti *et al.*, 1967; Sellakamur *et al.*, 1969) failed to demonstrate the induction of bladder cancer by 1-naphthylamine.

The purpose of the study reported here was to establish whether prolonged administration of purified 1-naphthylamine or mixtures of 1- and 2-naphthylamine similar in composition to early technical 1-naphthylamine could produce bladder cancer in dogs.

### METHODS AND MATERIALS

*Animals and experimental design.*—Beagle dogs bred in our own facility were used. Initially 18 males and 18 females, ~9 months old, were included in the experiment. After 75 months (June 1974) a further male and female, ~8 months old, were combined with the surviving 2 males and 1 female from the undosed control group to create a new group (Group 6) to which 2-naphthylamine was administered. The animals were distributed between 6 groups as in the Table in the next column.

| Group | Treatment (Daily, 5 days a week) | Dogs M | Dogs F | Origin |
|---|---|---|---|---|
| 1 | Control (lactose) | 4 | 4 | Initial group |
| 2 | 2 tablets (400 mg) purified 1-naphthylamine | 4 | 4 | Initial group |
| 3 | 2 tablets (400 mg) 1-naphthylamine containing 0·5% 2-naphthylamine | 4 | 4 | Initial group |
| 4 | 2 tablets (400 mg) 1-naphthylamine containing 6% 2-naphthylamine | 4 | 4 | Initial group |
| 5 | Undosed control | 2 | 2 | Initial group |
| 6 | 2 tablets (400 mg) pure 2-naphthylamine | 2 + 1 | 1 + 1 | Group 5 Additional dogs |

*Accommodation.*—Each dog was housed in a single kennel and had daily access to an exercise area. Each room contained 8 kennels and housed one group for the duration of dosing, except that in March 1975 the control dogs were rehoused for a period of 17 days in the rooms housing other groups. Cleaning and feeding equipment was confined to individual rooms and gloves, boots and aprons were worn by all staff on entering the rooms. The dogs were rehoused after dosing had ceased.

*Diet.*—All dogs received commercial dog diets, but one dog with signs of chronic nephritis was kept on a low-protein diet from the 75th month to the end of the experiment.

*Preparation and dosing of naphthylamines.*—Purified 1-naphthylamine (containing less than 100 pt/$10^6$ 2-naphthylamine) and pure 2-naphthylamine were supplied by Imperial Chemical Industries Limited, Organics Division, Blackley, Manchester, U.K. The two compounds were mixed in the appropriate proportions and tablets prepared from the following formulation:

| | mg |
|---|---|
| Naphthylamine or lactose | 200 |
| Spray-dried lactose | 274 |
| Microcrystalline cellulose | 120 |
| Magnesium stearate BP | 6 |
| Whole tablet | 600 |

Tablets were stored in a ventilated cabinet and dispensed into gelatin capsules for dosing. One gelatin capsule, containing 2 tablets of the appropriate formulation, was dosed orally to each dog in Groups 1, 2, 3, 4 and 6, daily for 5 days a week.

Groups 1, 2, 3 and 4 were started in March 1968 and dosing continued for 109 months, the experiment terminating in November

1978 after 128 months. Group 6 was constituted in June 1974 and dosing continued with 2-naphthylamine for 34 months. No dogs from Group 6 survived longer than 47 months from the start of dosing.

*Bioavailability of naphthylamines.*—Information on the dissolution of the naphthylamine tablets was obtained by estimating disintegration times in simulated gastric fluid (pH 1·5) in the standard B.P. test. Five or 6 tablets from 2 batches of each type were used.

*Observations.*—The dogs were observed daily, and any dogs with clinical symptoms requiring treatment were treated under veterinary supervision. In principle, treatment was aimed at prolonging the life of the dogs or alleviating painful conditions, but it was confined to treatment unlikely to affect the purpose and integrity of the study. Thus, treatment of wounds from fighting or the removal of dental tartar required general anaesthesia, and antibiotics were used to control various infections. Superficial tumours were removed surgically and examined histopathologically.

Dogs were weighed weekly for the first 13 weeks and thereafter at 4-weekly intervals. Blood samples were taken for clinical chemistry and haematology at 6-monthly intervals. The following parameters were estimated: haemoglobin, red-cell count and total and differential white-cell count, erythrocyte sedimentation rate, prothrombin time, kaolin-cephalin-time, methaemoglobin, plasma alkaline phosphatase (ALP), alanine transaminase (ALT) and ornithine carboxyl transferase (OCT) activities, plasma sodium and potassium, blood glucose and urea. During the 5th and 6th years of the experiment urine samples were collected by catheter for cytology.

*Pathology.*—Dogs which were moribund, and those that survived to the end of the experiment were killed with i.v. pentobarbitone and exsanguinated. All dogs were subjected to a full postmortem examination and tissues from up to 30 organs, and any organs with gross abnormalities, were taken for histopathology. After fixation in buffered formalin, tissues for histopathology were embedded in wax and 5 $\mu$m sections prepared. Sections were stained with haematoxylin and eosin for microscopy. Additional special stains were used on selected sections to aid diagnosis.

### RESULTS

### *Naphthylamine dosage*

The sample of 1-naphthylamine used for the experiment contained 5 pt/$10^6$ of 2-naphthylamine (nominal value, less than 100 pt/$10^6$). The sample of 2-naphthylamine contained no significant levels of impurities.

The average daily doses administered to the various groups are given in Table I. Each dog dosed at 400 mg per day for 109 months received 945 g of naphthylamine. The 4 dogs in Group 6, dosed for 34 months, received 290 g of 2-naphthylamine, Dog 35 from Group 6 died after 27 months and received $\sim$230 g. As the weight of the dogs changed with time, the group mean daily dose varied, but was usually within 3 mg/kg/day of the mean value calculated for the whole experiment.

Disintegration times of all tablets were in the range of $2\frac{1}{2}$–16 min except for one batch of 1-naphthylamine tablets where the range was 25–30 min.

### *Haematology*

Some individual values were abnormal, but there was no dose-related trend in any of the parameters measured. Dogs with haematuria showed moderate to marked anaemia.

### *Clinical chemistry*

There were no consistent trends in the control groups. Group 2 (1-naphthylamine) dogs had transient increases in plasma ALP, ALT and OCT activities, female 16 having marked increases from 7 years. Groups 3 and 4 had more frequent transient increases in plasma enzymes. Four of the 5 dogs in Group 6 had high plasma ALT activities, with less effect on plasma OCT. Plasma ALP activities were normal, while blood urea levels rose later in the study. No effects were observed on blood glucose or plasma K and Na.

### *Clinical observations*

A variety of conditions was observed in the dogs, including fits in some dogs and

TABLE I.—*Group mean daily doses of naphthylamines*

| Group | Compound(s) | Sex | Mean daily dose (mg/kg)* | Approximate mean daily dose of 2-naphthylamine (mg/kg) | Total dose of 2-naphthylamine (g) |
|---|---|---|---|---|---|
| 1 | — | | 0 | 0 | 0 |
| 2 | 1-naphthylamine | M | 18·4 | $9 \times 10^{-5}$† | |
| | | F | 22·0 | $1·1 \times 10^{-5}$† | $4·75 \times 10^{-6}$† |
| 3 | 1-naphthylamine + 0·5% 2-naphthylamine | M | 18·5 | 0·09 | |
| | | F | 21·1 | 0·11 | 4·75 |
| 4 | 1-naphthylamine + 6% 2-naphthylamine | M | 18·6 | 1·1 | |
| | | F | 20·9 | 1·25 | 56·7 |
| 6 | 2-naphthylamine | M | 18·4 | 18·4 | |
| | | F | 21·3 | 21·3 | 290 |

* Calculated as the mean daily dose administered 5 days per week to the 4 groups dosed naphthylamines:

$$\bar{x} = \frac{1}{T} \sum_{t=0}^{109} \frac{400}{\bar{x}_t}$$

where $\bar{x}$ = mean daily dose,
$\bar{x}_t$ = group mean weight at time t,
T = number of times dosed (in this case, 5 days per week).

† Based on 5 pt/$10^6$ of 2-naphthylamine in the purified 1-naphthylamine.

various conditions attributed to ageing. All dogs in Group 6, 1 in Group 4 and 1 in Group 2 suffered from haematuria for variable periods before they died or were killed in a moribund condition. In each case lesions of the bladder were observed at necropsy. No other treatment-related effects were found. The average body weights of treated male and female dogs were 15·3 and 13·5 kg respectively. No treatment-related effect on body weight was found.

*Urinary cytology*

An attempt to identify dogs with early bladder lesions by means of urinary cytology was unsuccessful. Similar problems had previously been reported (Radomski *et al.*, 1971).

*Pathology of the bladder*

The only organ to show gross lesions considered to be related to treatment was the urinary bladder. All 5 dogs in Group 6 (given 2-naphthylamine) had large cauliflower-like masses involving most of the bladder mucosa and nearly filling the lumen. Two dogs in Group 4 each had solitary papilliform masses protruding from the mucosa, the largest being 2 × 2 × 0·5 cm. Two dogs in Group 3 had a solitary 2mm diameter red nodule on the mucosal surface. One dog in Group 2 had a large blood clot in the bladder and the mucosa showed localized reddening.

The most significant histopathological finding was the transitional-cell carcinomas of the bladder in all 5 dogs receiving 2-naphthylamine (Fig. 1) and 2 early carcinomas in the 8 dogs receiving a mixture of 1-naphthylamine and 6% 2-naphthylamine. Two of the 8 dogs receiving 1-naphthylamine + 0·5% 2-naphthylamine had solitary haemangiomas arising in the submucosa and protruding into the bladder lumen. The 5 dogs in Group 6 developed bladder tumours within 25 to 47 months of dosing, whereas the bladder tumours in Groups 3 and 4 were detected at, or near to, the end of the study at 128 months.

One of the 8 dogs receiving purified 1-naphthylamine, which had shown haematuria during life, had focal cystitis with dilation of the submucosal blood vessels (Fig. 2) and associated localized haemorrhage. Focal epithelial hyperplasia of the wall of the bladder was seen in 2 dogs in the control group and 1 in Group 4. In addition to the bladder lesions, hyper-

TABLE II.—*Summary of*

| Organ | Histopathological diagnosis | 1 | 2 | 3 | 4 | 5 | 6 | 7 | 8 | 9 | 10 | 11 | 12 | 13 | 14 | 15 | 16 |
|---|---|---|---|---|---|---|---|---|---|---|---|---|---|---|---|---|---|
| | Group | 1 (Lactose) | | | | | | | | 2 (1-naphthylamine) | | | | | | | |
| | Number of dog | 1 | 2 | 3 | 4 | 5 | 6 | 7 | 8 | 9 | 10 | 11 | 12 | 13 | 14 | 15 | 16 |
| | Sex of dog | M | | | | F | | | | M | | | | F | | | |
| | Time to death (months) from start of dosing | 24 | 128 | 95 | 125 | 122 | 128 | 84 | 128 | 120 | 128 | 127 | 54 | 18 | 100 | 126 | 109 |
| Bladder | Haemangioma | | | | | | | | | | | | | | | | |
| | Early carcinoma | | | | | | | | | | | | | | | | |
| | Transitional-cell carcinoma | | | | | | | | | | | | | | | | |
| Aorta | Chemodectoma | | | | | | | | | | | | | | | | |
| Gut | Leiomyoma | | | | × | | | | | | | | | | | | |
| | Lymphosarcoma | | | | | | | | | × | | | | | | | |
| Adrenal | Cortical adenoma | | | | | | | | × | | | | | | | | |
| Gall bladder | Submucosal fibroma | | | | | | | | | | | | | | × | | |
| Liver | Cholangiocarcinoma | | | | | | | | | | | | | | | | |
| Pancreas | Acinar adenoma | | | | | | | | | | | | | | × | | |
| Pituitary | Basophil adenoma | | | | | | | | × | | | | | | | | |
| Thyroid | Follicular adenoma | | × | | | | × | | | | | | | | | | |
| | Parafollicular adenoma | | | | | | | | | | | | | | | | |
| | Carcinoma | | | | | | | | | | | × | | | | | × |
| Parathyroid | Adenoma | | | | | | | | | | | | | | | | |
| Spleen | Fibrosarcoma | | | | | | | | | | | × | | | | | |
| Eye | Melanoma of iris | | | | | | | | | | | | | | | | |
| Skin | Lipoma | | × | | | | | | | | × | | | | | × | |
| | Haemangioma | | | | | ×[1] | | | | | | | | | | | |
| | Papilloma | | | | | | | | | | | | | | | | × |
| | Keratoacanthoma | | | | | | | | | | × | | | | | ×[12] | |
| | Histocytoma | | | | | | | | | | | | | | | | |
| | Haemangio-pericytoma | | | | | | | | | | | | | | | | |
| | Apocrine carcinoma | | | | | | | | | | | | | | ×[4] | | |
| | Squamous carcinoma | | | | | | | | | ×[5] | | | | | | | |
| | Mast-cell tumour | | | | | | | | | | | | | | | | |
| Nasal cavity | Fibrosarcoma | | | | | | | | | | | | | | | | |
| Oral cavity | Epulis | | | | | | | | | | × | | | | | | |
| Ovary | Granulosa-cell tumour | | | | | | | | | | | | | | | | |
| | Adenoma | | | | | × | | | | | | | | | | | |
| Vagina | Fibroma | | | | | | | | | | | | | | | | |
| Mammary gland | Mixed malignant tumour | | | | | ×[9] | ×[10] | | | | | | | | | | |
| | Duct carcinoma | | | | | | | | | | | | | | | | |
| Testis | Seminoma | | | | | | | | | | | | | | | | |
| | Sertoli-cell tumour (bilateral) | | | | | | | | | | | | | | | | |

Reference numbers within the table

1 103 months
2 113 months
3 106 months
4 97 months
5 100 months
6 124 and 126 months
7 109 months
8 115 months
9 4 tumours: 106, 109, 115 months and at termination (128 months)

plasia of the epithelium of the ureter was seen in single dogs in Groups 3 and 4 and 3 dogs in Group 6.

*Pathology of other organs*

A variety of gross lesions was observed at postmortem examination of the dogs in all groups. Histopathological examination revealed a range of conditions, many of which (*e.g.* interstitial nephritis, nodular hyperplasia of the spleen and liver, hepatic granulomata and inflammatory changes in the respiratory tract) are recognized as common in ageing dogs. In none except for those of the urinary tract was there a marked difference in incidence from the control group, and they were not considered to be caused by treatment with naphthylamines.

*tumour incidence*

| 3 (1-naphthylamine + 0·5% 2-naphthylamine) | | | | | | | | 4 (1-naphthylamine + 6% 2-naphthylamine) | | | | | | | | 6 (2-naphthylamine) | | | | | 5 Control |
|---|---|---|---|---|---|---|---|---|---|---|---|---|---|---|---|---|---|---|---|---|---|
| 17 | 18 | 19 | 20 | 21 | 22 | 23 | 24 | 25 | 26 | 27 | 28 | 29 | 30 | 31 | 32 | 33 | 34 | 35 | 37 | 38 | 36 |
| M | | | | F | | | | M | | | | F | | | | M | M | F | M | F | F |
| 17 | 128 | 121 | 128 | 122 | 68 | 128 | 108 | 124 | 128 | 66 | 125 | 128 | 128 | 125 | 96 | 44 | 38 | 27 | 47 | 35 | 28 |
| | | | × | | | × | | | | | | | | | | | | | | | |
| | | | | | | | | | | | | | × | × | | | | | | | |
| | | | | | | | | | | | | | | | | × | × | × | × | × | |
| | | | | | | | | | | | × | | | | | | | | | | |
| | | | | | | | | | | | | | × | × | | | | | | | |
| | | | | × | | | | | | | | | | | | | | | | | |
| | | | × | | | | | | × | | | | × | | | | | | | | |
| | × | | | | | | | | | | | | | | | | | | | | |
| | | | | | | | | | | | × | | | × | × | | | × | | | |
| | | | | | | | | | | | | | | × | | | | | | | |
| | | × | | | | | | | | | × | | | | | × | | | | | |
| | | | | | | | | | | | | | × | | | | | | | | |
| | | | | | | × | | | | | | × | × | | | | | | | | |
| | × | | × | | | × | | | | | | | | | | | | | | | |
| | | | ×[1] | | | | | | | | | | | | | | | | | | |
| | | | × | | | | | | | | | | ×[3] | | | | | | | | |
| | | | | | | | | | | | | | ×[8] | | | | | | | | |
| | | | ×[6] | | | | | | | | | ×[7] | | | | | | | | | |
| | | | | | | | | | | | ×[2] | | | | | | | | | | |
| | | | | | × | | | | | | | | | | | | | | | | |
| | | | | | | | | | | | | | | × | | | | | | | |
| | | | | × | | | | | | | | | | | | | | | | | |
| | | | | | | | | | | | | | × | | | | | | | | |
| | × | | × | | | | | | | | | | | | | | | | | | |
| | | | | | | | | | ×[11] | | | | | | | | | | | | |

[10] 82 months
[11] 119 months and at termination (128 months)
[12] 88 months

Benign or malignant tumours were diagnosed in 29 dogs (Table II). Twelve tumours were removed surgically during the course of the experiment from 10 dogs. Nine of these were from the skin or subcutis, 2 from the mammary gland and 1 from the testis. The diagnosis of each tumour is given in Table II.

The number of tumour-bearing animals for each group is given in Table III. The only statistically significant differences in incidence were the increased incidence of bladder tumours ($P < 0\cdot01$) and of malignant-tumour-bearing animals ($P < 0\cdot05$) in Group 6 (2-naphthylamine) over the controls. There was no significant difference between tumour incidence in Groups 2, 3 and 4 and controls.

FIG. 1.—Transitional-cell carcinoma of the bladder in a dog which had received 2-naphthylamine for 47 months, showing extensive invasion of the muscle of the bladder wall. H. & E. ×270.

FIG. 2.—Bladder of a dog which had received purified 1-naphthylamine for 109 months. There are several large dilated blood vessels in the submucosa and moderate inflammatory-cell infiltration. H. & E. ×105.

### DISCUSSION

Unlike many of the previous studies on the carcinogenicity of naphthylamines, the purity of the chemicals administered in this study was high. The contamination of 1-naphthylamine by 2-naphthylamine (5 pt/$10^6$) meant extremely small doses of 2-naphthylamine to the dogs in Group 2.

TABLE III.—*Number of tumour-bearing animals and tumours in each group*

| Group | 1 | 2 | 3 | 4 | 6 |
|---|---|---|---|---|---|
| No. of animals* | 7 | 7 | 7 | 8 | 5 |
| No. of malignant TBA | 2 | 4 | 4 | 5 | 5 |
| No. of benign TBA | 3 | 2 | 2 | 1 | 0 |
| Total TBA | 5 | 6 | 6 | 6 | 5 |
| Total malignant tumours | 2 | 6 | 4 | 8 | 7 |
| Total benign tumours | 8 | 8 | 13 | 14 | 0 |
| Total tumours/group | 10 | 12 | 17 | 22 | 7 |

* No. of animals surviving longer than 50 months, except for Group 6, where there were no survivors after 47 months.
TBA = Tumour-bearing animals.

The time taken for the naphthylamine tablets to disintegrate was within the specified maximum disintegration times for uncoated tablets, except for one batch of the 1-naphthylamine tablets. The effect of this slow disintegration time on bioavailability is difficult to assess accurately, but assuming that the batches examined were representative of all batches made, it is likely to have delayed absorption without affecting the total amount absorbed.

Transient increases in plasma enzyme levels, which were most marked in dogs on pure 2-naphthylamine, may have been associated with naphthylamine treatment, though no statistical treatment of the results is possible. The marked increases in plasma ALT activity were not associated with pathological changes in the liver, and their cause is unexplained. High blood urea levels are to be expected in dogs with cancer of the urinary tract.

The induction of malignant tumours of the bladder in the 5 dogs receiving 2-naphthylamine confirms the carcinogenicity of this compound. Similarly, the induction of a low incidence of bladder cancer in the group receiving 1-naphthylamine with 6% added 2-naphthylamine provides an explanation for the carcinogenic effect of impure 1-naphthylamine in early carcinogenicity and epidemiology studies. The significance of haemangiomas of the bladder in 2 of the dogs receiving 1-naphthylamine + 0·5% 2-naphthylamine is uncertain, as similar lesions do not appear to have been reported in previous studies with naphthylamines in the dog. According to Moulton (1978), haemangioma and haemangiosarcoma account for ~6% of all naturally occurring bladder neoplasms in domestic animals.

The absence of bladder cancer in the dogs receiving 1-naphthylamine is evidence of its non-carcinogenicity. Previous studies (Gehrmann *et al.*, 1949; Radomski *et al.*, 1980) which were limited, for example by the absence of control groups, were also negative. In this study large doses were given over a prolonged period of time and both negative and positive controls were used. Although the number of animals was relatively small (8 per group) from a statistical point of view, our study is the largest study reported on amine carcinogenesis in dogs, and was initiated at a time when group sizes, even in rodent studies, were relatively small. Thus in spite of the inadequacies resulting from small group sizes, this experiment provides the best data so far available on 1-naphthylamine carcinogenesis in dogs.

One dog in the group receiving 1-naphthylamine developed haematuria. The localized vascular dilation observed in the bladder submucosa may represent a pre-angiomatous change, but cannot be considered neoplastic. No similar lesion was observed in the other groups receiving naphthylamine, so it is unlikely to be treatment-related.

An experiment of this size, with a limited number of animals in each group, may be considered inadequate to define the non-carcinogenicity of 1-naphthylamine. Indeed, proof of non-carcinogenicity is always impossible, but a comparison of the doses of naphthylamines administered gives an indication of the minimum difference in potency between the 2 compounds. If the 2 haemangiomas of the bladder in Group 3 are taken as an indication of the activity of 2-naphthylamine, the minimum dose of 2-naphthylamine to produce an effect is about 0·1 mg/kg/day (Table I). This is 0·5% of the dose of 1-naphthylamine given to Group 2; hence 1-naphthylamine can be considered to be at least 200 × less potent than 2-naphthylamine in inducing bladder cancer in dogs. If the much shorter latent period in the group receiving pure 2-naphthylamine is taken into account, the potency difference can be considered to be even greater.

A large variety of other tumours was seen in this experiment. Larger numbers of tumours of the thyroid and skin were observed in the naphthylamine-treated groups (2, 3 and 4) than in the control group, but the difference was not significant.

The carcinogenicity of 2-naphthylamine is attributed to its metabolism in the liver to the N-hydroxy and -nitroso derivatives, which are excreted in the urine either as conjugates or as the metabolite itself. The conjugates are unstable in the acidic condition in the bladder, and hydrolyse yielding the original oxidation products (Radomski *et al.*, 1971).

In the case of both 1- and 2-naphthylamine the N-hydroxy derivatives are carcinogenic. It is thus not surprising that both 1- and 2-naphthylamine are positive in *in vitro* mutagenicity assays which incorporate liver microsomal preparations for metabolism (de Serres & Ashby, 1981). The N-hydroxy derivative of 1-naphthylamine is excreted in the urine (Deichmann & Radomski, 1969), is considered stable and unreactive with low-mol.-wt urinary nucleophils (Kadlubar *et al.*, 1978) and is more potent at sites of direct application than the 2-hydroxy derivative (Radomski *et al.*, 1971; Kadlubar *et al.*, 1978).

The reason for the absence of a carcinogenic effect of 1-naphthylamine may, however, relate to the quantity of active metabolites excreted in the urine. There is a quantitative correlation between the carcinogenicity of these compounds to dog bladder and the combined excretion of N-hydroxy and N-nitroso metabolites (Clayson & Garner, 1976). However, there is only about a 40-fold difference in the quantity of metabolites excreted after a dose of 5 mg/kg (0·8 μg for 1-naphthylamine and 30 μg for 2-naphthylamine (Radomski *et al.*, 1971)) and at the higher doses used in our experiment this difference may even be smaller. This quantitative difference in the metabolism of 1- and 2-naphthylamine is reflected in a similar difference in the level of reaction with DNA in the urothelium. DNA–naphthylamine adducts could be detected in the urothelium (18·5 adducts/$10^8$ nucleotides) 2 days after administration of 2-naphthylamine to a dog; but none were detected after administration of 1-naphthylamine (Kadlubar *et al.*, 1981). Because the limit of detection of adducts was 1/$10^8$ nucleotides, the magnitude of the difference can only be expressed as at least 18-fold. More precision in the estimation of the DNA adducts could provide an estimate of the difference in potency between the naphthylamine isomers. Comparison with *in vivo* data and the levels of excretion of metabolic products would be a further step in understanding the reason for the absence of carcinogenic effect of 1-naphthylamine.

Many people have contributed to this study during the last 11 years, and the continued interest and support of ICI Organics Division in this project was crucial to its successful outcome. Thanks are due to Dr D. C. Taylor for determining disintegration times, and to Mrs I. Stevenson for skilled technical assistance.

### REFERENCES

Bonser, G. M. (1943) Epithelial tumours of the bladder in dogs induced by pure β-naphthylamine. *J. Pathol. Bacteriol.*, **55**, 1.

Bonser, G. M., Clayson, D. B., Jull, J. W. & Pyrah, L. N. (1952) The carcinogenic properties of 2-amino-1-naphthol hydrochloride and its parent amine 2-naphthylamine. *Br. J. Cancer*, **6**, 412.

Bonser, G. M., Clayson, D. B., Jull, J. W. & Pyrah, L. N. (1956) The carcinogenic activity of 2-naphthylamine *Br. J. Cancer*, **10**, 533.

Case, R. A. M., Hosker, M. E., McDonald, D. B. & Pearson, J. T. (1954) Tumours of the urinary bladder in workmen engaged in the manufacture and use of certain dyestuff intermediates in the British chemical industry. I. The role of aniline, benzidine, alpha-naphthylamine and beta-naphthylamine. *Br. J. Industr. Med.*, **11**, 75.

CLAYSON, D. B. & GARNER, R. C. (1976) Carcinogenic aromatic amines and related compounds. In *Chemical Carcinogens, ACS Monograph* **173** (Ed. C. E. Searle) Washington DC: American Chemical Society. p. 366.

CONZELMAN, G. M., JR & MOULTOX, J. E. (1972) Dose–response relationships of the bladder tumorigen 2-naphthylamine: A study in beagle dogs. *J. Natl Cancer Inst.*, **49**, 193.

CONZELMAN, G. M., JR, MOUTLON, J. E., FLANDERS, L. E. III, SPRINGER, K. & CROUT, D. (1969) Induction of transitional-cell carcinomas of the urinary bladder in monkeys fed 2-naphthylamine. *J. Natl Cancer Inst.*, **42**, 825.

DEICHMANN, W. B. & RADOMSKI, J. L. (1969) The carcinogenicity and metabolism of aromatic amines in the dog. *J. Natl Cancer Inst.*, **43**, 263.

GERHMANN, G. H., FOULGER, J. H. & FLEMING, A. J. (1949) Occupational carcinoma of the bladder. In *Proc. IX Int. Cong. Indust. Med.* Bristol: Wright. p. 427.

GOLDWATER, L. J., ROSSO, A. J. & KLEINFELD, M. (1965) Bladder tumors in a coal-tar dye plant. *Arch. Environm. Hlth*, **11**, 814.

HADIDIAN, Z., FREDRICKSON, T. N., WEISBURGER, E. K., WEISBURGER, J. H., GLASS, E. M. & MANTEL, N. (1968) Tests for chemical carcinogens. Report on the activity of derivatives of aromatic amines, nitrosamines, quinolines, nitroalkanes, amides, epoxides, aziridines and purine antimetabolites. *J. Natl Cancer Inst.*, **41**, 985.

HICKS, R. M., CHOWANIEC, J. & WAKEFIELD, J. St J. (1978) Experimental induction of bladder tumours by a two-stage system. In *Carcinogenesis, Vol. 2 Mechanisms of Tumour Promotion and Carcinogenesis.* Eds: Slaga *et al.* New York: Raven Press. p. 45.

HUEPER, W. C., WILEY, F. H. & WOLFE, H. D. (1938) Experimental production of bladder tumors in dogs by administration of beta-naphthylamine. *J. Induts. Hyg.*, **20**, 46.

KADLUBAR, F. F., ANSON, J. F., DOOLEY, K. L. & BELAND, F. A. (1981) Formation of urothelial and hepatic DNA adducts from the carcinogen 2-naphthylamine. *Carcinogenesis*, **2**, 467.

KADLUBAR, F. F., MILLER, J. A. & MILLER, E. C. (1978) Guanyl $0^6$-acrylamination and $0^6$-arylation of DNA by the carcinogen N-hydroxy 1-naphthylamine. *Cancer Res.*, **38**, 3628.

MOULTON, J. E. (1978) Tumors of the urinary system. In *Tumors in Domestic Animals, 2nd edn.* Ed. Moulton. California: University Press. p. 302.

MANCUSO, T. F. & EL-ATTAR, A. A. (1967) Cohort study of workers exposed to beta-naphthylamine and benzidine. *J. Occup. Med.*, **9**, 277.

RADOMSKI, J. L., BRILL, E., DEICHMANN, W. B. & GLASS, E. M. (1971) Carcinogenicity testing of N-hydroxy and other oxidation and decomposition products of 1- and 2-naphthylamine. *Cancer Res.*, **31**, 1461.

RADOMSKI, J. L., DEICHMANN, W. B., ALTMAN, N. H. & RADOMSKI, T. (1980) Failure of pure 1-naphthylamine to induce bladder tumors in dogs. *Cancer Res.*, **40**, 3537.

SAFFIOTTI, U., CEFIS, F., MONTESANO, R. & SELLAKAMUR, A. R. (1967). Induction of bladder cancer in hamsters fed aromatic amines. In *Bladder Cancer: A Symposium.* Ed. Deichmann & Lampe. Birmingham, Alabama: Aesculapius. p. 129.

SELLAKAMUR, A. R., MONTESANO, R. & SAFFIOTTI, U. (1969) Aromatic amines carcinogenicity in hamsters. *Proc. Am. Ass. Cancer Res.*, **10**, 78.

DE SERRES, F. J. & ASHBY, E. J. (eds) (1981) Short-term tests for carcinogens. *Report of the International Collaborative Program.* Amsterdam: Elsevier/North Holland. p. 130.